# Cerebrolysin treatment in a case of post-herpes simplex encephalitis with neuropsychiatric symptoms

**DOI:** 10.25122/jml-2025-0052

**Published:** 2025-09

**Authors:** Young Seok Lee, Jinmi Seo, Soo Hwan Yim

**Affiliations:** 1 Department of Neurology, Gangneung Asan Hospital, College of Medicine, Ulsan University, Gangneung, Republic of Korea

**Keywords:** herpes simplex encephalitis, neuropsychiatric symptoms, Cerebrolysin, case report

## Abstract

Herpes simplex encephalitis (HSE) is a severe central nervous system (CNS) infection associated with cognitive decline, psychotic symptoms, and epilepsy. Despite aggressive antiviral and corticosteroid treatments, post-HSE complications remain challenging to manage. We report a case of a 33-year-old female patient with post-HSE neuropsychiatric symptoms who showed significant improvement following Cerebrolysin treatment. The patient was treated with acyclovir and dexamethasone in the acute phase, followed by Cerebrolysin administration in the post-acute phase. This case highlights the potential therapeutic role of Cerebrolysin in managing post-encephalitic cognitive dysfunction, underscoring the need for further studies.

## Introduction

Herpes simplex encephalitis (HSE) is a common cause of viral encephalitis that can lead to severe neurological and neuropsychiatric sequelae, including cognitive impairment, epilepsy, and behavioral abnormalities [1,2]. Standard treatment with intravenous acyclovir and corticosteroids mitigates acute infection but does not prevent long-term complications [3,4]. Currently, no established treatments exist for managing post-HSE complications. Cerebrolysin is a neuropeptide preparation with potential neurorestorative effects [5]. Cerebrolysin has shown promise in experimental models of neuroregeneration [6]. However, clinical evidence in post-encephalitic patients remains limited. This report describes a case that demonstrated a clinically meaningful benefit from Cerebrolysin administration.

## Case presentation

A 33-year-old female with no prior medical history presented to the emergency department with fever (38.4°C), headache, and anterograde amnesia. The patient reported upper respiratory symptoms for 5 days prior to admission. Initially, she presented with fever and voiding difficulty, leading to a misdiagnosis of cystitis at another hospital. However, despite receiving treatment for a urinary tract infection, the patient's symptoms progressed, and on day 5 of symptom onset, she was transferred to our hospital due to altered consciousness. Upon arrival at the emergency department, routine blood tests and urine analysis were performed; however, no significant abnormalities were found to explain the fever and impaired consciousness. Consequently, a lumbar puncture was performed to investigate the possibility of a central nervous system (CNS) infection or other neurological disorders.

**Figure 1:**
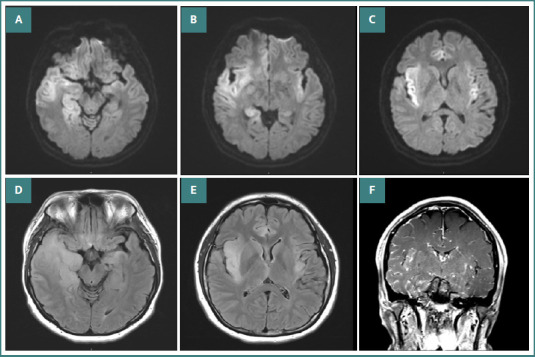
Brain MRI diffusion-weighted images. A–C indicated a diffuse cortical diffusion restriction lesion centered on the insular region. Brain MRI FLAIR images; D–E also demonstrated this finding. Additionally, brain MRI with gadolinium enhancement (F) suggested focal cerebral edema and a diffuse inflammatory lesion in the right temporal and left insular regions.

**Figure 2:**
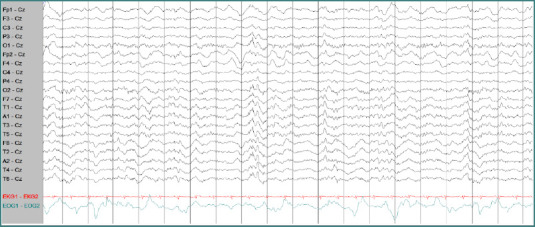
The initial EEG showed continuous periodic lateralized epileptiform discharges at 0.3 Hz in the right temporal region, first appearing one minute after the second period of closed eyes. This EEG was obtained five minutes after the second period of eyes closed with awake.

Cerebrospinal fluid (CSF) analysis revealed an elevated white blood cell (WBC) count (65 cells/µL; lymphocytes 80%, monocytes 14%, neutrophils 5%, atypical cells 1%), red blood cells (RBCs) 15 cells/µL, protein 97.8 mg/dL, and glucose 65 mg/dL (with corresponding serum glucose of 130 mg/dL). Herpes simplex virus type 1 (HSV-1) DNA was detected in the CSF, confirming the diagnosis of herpes simplex encephalitis (HSE). The clinical course is summarized in Table 1. Brain magnetic resonance imaging (MRI) demonstrated a diffuse cortical diffusion restriction lesion centered on the insular region (Figure 1A-F). Electroencephalography (EEG) showed continuous periodic lateralized epileptiform discharges (PLEDs) at 0.3 Hz in the right temporal region, first appearing one minute after the second period of eye closure (Figure 2A-C).

**Table 1:** Timeline of the patient’s clinical course, diagnostic findings, and therapeutic interventions during hospitalization and follow-up

Timeline	Clinical Course & Interventions
**Day 0**	**First Admission** Fever, Urinary symptoms, Confusion, Anterograde Amnesia Acyclovir 10mg/kg three times daily (14 days) Dexamethasone 2mg four times daily (4 days) MMSE 18/30
**Day 7**	**HSV-1 Confirmed in CSF** CSF: WBC 65(Lymphocyte 80%, monocyte 14%), Protein 97.8 MRI: Right temporal involvement EEG: Periodic epileptiform discharges in the right temporal region
**Day 30**	**Discharged** Cognitive impairment persists (MMSE 22/30) New symptoms: Agitation, Behavioral changes
**Day 45**	**Second Admission** Worsening neuropsychiatric symptoms Dysphagia present
**Day 50**	**Cerebrolysin Treatment** 10 mL BID for 1 week, then 10 mL TID for 3 weeks Initial response minimal
**Week 14**	**Gradual Improvement** MMSE increased 26/30 Behavioral symptoms improved
**Month 6**	**Full Recovery** MMSE 30/30 Return to work (Bakery) MRI: Residual right temporal atrophy EEG: Normalized rhythm

BID, twice daily; TID, three times daily

The patient received intravenous acyclovir (10 mg/kg every 8 hours) for two weeks, which led to an improvement in urinary frequency and urgency. Cognitive function also showed partial improvement, with the Mini-Mental State Examination (MMSE) score increasing from 18 to 22. However, persistent amnesia and new-onset dysphagia were observed one month after discharge. The patient was subsequently readmitted due to worsening neuropsychiatric symptoms and progressive swallowing dysfunction, characterized by food retention in the mouth without swallowing. Despite the initial improvement following treatment for herpes simplex encephalitis, neurological and psychiatric manifestations gradually worsened after discharge. The patient developed disinhibited sexual behavior and exhibited episodes of irritability, verbal aggression, and physical aggression. The patient's family reported increasing agitation, confusion, and behavioral changes, which significantly impaired daily functioning.

Given the worsening neuropsychiatric symptoms and swallowing difficulties, further evaluation and management were deemed necessary. A psychiatric consultation was performed, and considering the temporal association of symptom onset, the findings strongly suggested that the neuropsychiatric manifestations were attributable to herpes simplex encephalitis.

**Figure 3:**
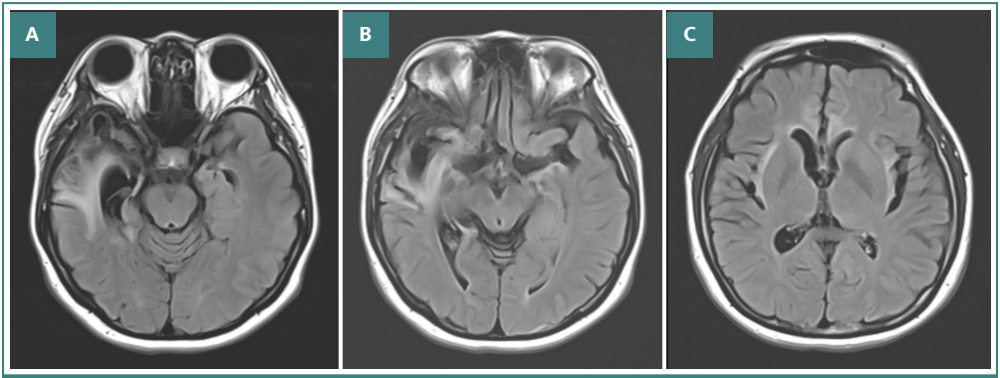
Second Brain MRI (FLAIR) after 6 months showed cerebromalacia in the right anterior temporal region combined with a dilated right ventricle horn (A-C).

**Figure 4:**
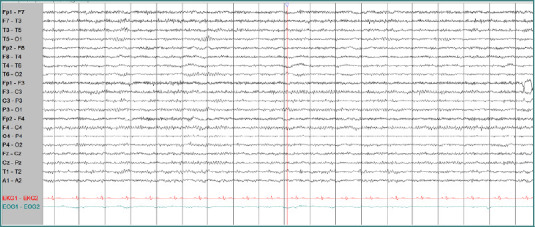
Follow-up EEG revealed normal background activity without definitive epileptiform discharges. This EEG was obtained three minutes after the second period of eyes closed with awake.

The patient received intravenous Cerebrolysin infusion [7]. The treatment regimen consisted of 10 mL of Cerebrolysin diluted in 100 mL of saline solution, administered intravenously twice daily during the first week and subsequently increased to three times daily, with a total daily dose of 30 mL. No adverse effects or complications were observed during the treatment period. Initial clinical improvements were minimal. However, after 4 weeks of Cerebrolysin therapy, the patient exhibited gradual improvement in both cognitive and behavioral symptoms. Throughout the 4-week course of Cerebrolysin treatment, the patient underwent only basic motor rehabilitation and did not receive any additional specialized physical or speech therapy. Over time, cognitive function, neuropsychiatric symptoms, and motor abilities steadily improved. At 6 months post-treatment, the patient's MMSE score recovered to 30, indicating a return to normal cognitive function. The patient was able to return to work as a baker and successfully resumed operating her own bakery. The patient reported that before treatment, she had significant difficulties managing her daily life and felt hopeless about returning to work. After undergoing Cerebrolysin therapy, she gradually regained confidence and cognitive function. Follow-up brain MRI (6 months post-treatment) showed residual cerebromalacia in the right anterior temporal region with a dilated right ventricular horn (Figure 3). Follow-up EEG demonstrated a normalized background rhythm without epileptiform discharges (Figure 4).

The patient reported: ‘*At first, I was afraid I would never be able to bake again*,’ reflecting her initial distress and later satisfaction with the treatment outcome. She expressed great satisfaction with the treatment and was relieved to regain independence in her personal and professional life.

## Discussion

This case suggests that Cerebrolysin may contribute to long-term neurological recovery, particularly in patients experiencing neuropsychiatric and functional impairments following HSE. Several clinical and animal studies have investigated treatments targeting brain swelling due to neuroinflammation following HSE. Corticosteroid therapies, including dexamethasone (used in our case), hydrocortisone, and methylprednisolone, have shown relatively positive outcomes. Moreover, experimental animal studies have explored the use of toll-like receptor agonists, interferon-alpha, and intravenous immunoglobulin as potential immunomodulatory treatments [4]. These approaches primarily aim to prevent complications arising from neuroinflammation. To date, no clinical studies have been published confirming the efficacy of Cerebrolysin in viral encephalitis. However, based on its previously demonstrated positive effects in various CNS disorders [8-11], it is reasonable to hypothesize that Cerebrolysin could provide therapeutic benefits in viral encephalitis as well.

The potential efficacy of Cerebrolysin in HSE can be considered in three key aspects. First, it exhibits anti-inflammatory properties, which may be particularly relevant in viral encephalitis, where neuroinflammation plays a critical role in disease progression and long-term complications [12]. Neuroinflammation following viral encephalitis triggers cytokine secretion and immune cell recruitment, leading to neurological complications, cognitive and memory impairment, and psychiatric disorders such as depression. This is why corticosteroids are often administered in conjunction with antiviral agents to control neuroinflammation [4]. Notably, Cerebrolysin has been shown to modulate microglial activation in experimental autoimmune encephalomyelitis models, an effect that may also be relevant in viral encephalitis [6]. Preclinical studies have demonstrated that Cerebrolysin reduces inflammatory cytokines, including interleukin-1β (IL-1β), tumor necrosis factor-α (TNF-α), and interleukin-6 (IL-6), while suppressing microglial activation, as evidenced by a reduction in ionized calcium-binding adaptor molecule 1 (Iba1) expression.

Second, Cerebrolysin exerts neuroprotective effects by mimicking the activity of endogenous neurotrophic factors (NTFs), enhancing the levels of brain-derived neurotrophic factor (BDNF), nerve growth factor (NGF), and glial cell line-derived neurotrophic factor (GDNF)[5]. By increasing these neurotrophic factors, neuronal survival may be promoted, synaptic connectivity enhanced, and neuronal apoptosis reduced. Since HSE and other forms of viral encephalitis can lead to progressive neurodegeneration, these neuroprotective properties suggest that Cerebrolysin could help preserve neuronal function and structure during and after viral CNS infections.

Third, Cerebrolysin has been well-documented to enhance neuroplasticity and promote neuroregeneration, particularly in conditions such as stroke and traumatic brain injury [9]. Demonstrated benefits of Cerebrolysin in stroke, post-traumatic brain injury, and subarachnoid hemorrhage (SAH) include the improvement of neurological deficits and the reduction of long-term disability [10]. In patients with stroke and National Institutes of Health Stroke Scale (NIHSS) scores of 12 or higher, Cerebrolysin has been shown to significantly improve functional recovery [8]. This is thought to be due to increased synaptic formation and promotion of dendritic growth. Cerebrolysin is generally well tolerated, with no reports of life-threatening adverse effects in previous clinical studies [9]. However, it should be used with caution in patients with renal impairment, and dose adjustments may be necessary in such cases. Given these mechanisms, it is plausible that Cerebrolysin could aid in the recovery of neurological deficits following viral encephalitis, particularly in improving motor and cognitive function.

Post-HSE neuropsychiatric complications significantly impact patients' quality of life, yet treatment options remain limited. This case suggests Cerebrolysin may have a role in improving cognitive and psychiatric sequelae of post-HSE. The mechanism of action of Cerebrolysin involves neurotrophic support, neuronal protection, and enhancement of synaptic plasticity, which may contribute to its efficacy in post-infectious encephalopathy. While the clinical improvement observed in this case is promising, large-scale studies are necessary to establish the efficacy and optimal regimen of Cerebrolysin for post-HSE complications.

## Conclusion

This case highlights the potential benefit of Cerebrolysin in treating cognitive and neuropsychiatric dysfunction following HSE. Given its strong safety profile and neuroprotective properties, Cerebrolysin could be considered an adjunctive therapy for HSE, particularly in preventing long-term neurological sequelae. Given the lack of established treatments for post-HSE complications, further research is warranted to explore the neuroprotective and regenerative effects of Cerebrolysin in this patient population.

## Data Availability

For full clinical, MRI, and EEG data, kindly refer to the corresponding author (Soo Hwan Yim).
